# Fabrication and characterization of enhanced hydrazine electrochemical sensor based on gold nanoparticles decorated on the vanadium oxide, ruthenium oxide nanomaterials, and carbon nanotubes composites

**DOI:** 10.3906/kim-2009-58

**Published:** 2021-08-27

**Authors:** Sibel KARACA, Süleyman KOÇAK

**Affiliations:** 1 Manisa Celal Bayar University, Science and Art Faculty, Department of Chemistry, Manisa Tukey; 2 Manisa Celal Bayar University Applied Science Research Center (DEFAM), Manisa Tukey

**Keywords:** mixed vanadium oxide and ruthenium oxide, gold nanoparticles, hydrazine, pulsed deposition, amperometry

## Abstract

This work describes the synthesis of mixed oxide film of vanadium and ruthenium by pulsed deposition technique on multiwall carbon nanotubes and the decoration of gold nanoparticles on the mixed film. A ternary electrocatalyst has been developed for the electrochemical oxidation of hydrazine by combining two metal oxide mixtures with Au nanoparticles. Surface morphology and chemical composition of the electrode have been examined with SEM, EDX, HRTEM, EIS, and XRD. The peak current of hydrazine increased 9 times at the AuNPs/(VOx-RuOx)/CNT/GCE compared to the bare GCE, and the peak potential shifted to negative 848 mV. Linear sweep voltammetry (LSV) and amperometric techniques revealed that the AuNPs/(VOx-RuOx)/CNT/GCE displays linear concentration range 2.5–10000 µM (LSV) and the concentration range 0.03–100 µM (amperometry). The limit of detection (LOD) is 0.5 μM and 0.1 μM at (S/N = 3) for LSV and amperometric technique, respectively. The results obtained show a good RSD% of 2.1%–3.2% and reasonable recovery of 97%–108% of hydrazine detection.

## 1. Introduction

Hydrazine can be used as textile dyes, drugs, fuel cells, pesticides, foaming agents, blowing agents, emulsifiers, fuels in spacecraft, and corrosion inhibitors. The Environmental Protection Agency (EPA) defines hydrazine as a toxic chemical, and hydrazine should not exceed 1 ppm in commercial wastewater. It is of great importance to develop accurate and sensitive methods of determining the amount of hydrazine for purposes of environmental protection and maintenance of human health. For the detection of hydrazine, different analytical methods have been reported, in the literature, such as chemiluminescence [1], spectrophotometric [2], colorimetric method [3], fluorimetric sensor [4], chromatography [5], titrimetric, and electrochemical techniques [6]. Electrochemical techniques are privileged compared to other techniques thanks to their unique advantages such as high sensitivity and selectivity, low cost, and quick response [7–9]. However, the electrooxidation of hydrazine in bare carbon electrodes has a high overpotential and a slow reaction. Surface modification of the carbon electrode with different nanomaterials is promising for reducing the high overpotential [10–12]. Accordingly, it can be modified with various electrode materials such as carbon-based materials [13,14], conductive polymers [15–18], organic dyes [19], metal oxides [20,21], and metal nanoparticles [7,11,19,22,23] for detection of analytes.

Some metal oxides such as Co_3_O_4 _[20], CuO [24], VO_2_, Cr_2_O_3_, MnO_3 _[25], FeCo oxides [26], ZnO [27], and Fe_2_O_3_/CeO_2_ nanocubes [28] are used as a modifier of electrodes for the oxidation of hydrazine. Also, metal oxides and their double and triple combinations are promising compounds for electrochemical applications [29,30]. Nanostructured metal oxides have been evaluated in various energy devices such as fuel and solar cells, Li-ion batteries, and supercapacitors. Mixed metal oxides exhibit stoichiometric or nonstoichiometric compositions [31]. Electrocatalytic activity can be explained by the interaction between nanostructured metal particles (hyper) and metal oxide (hypo) composites, known as the “hypo-hyper-d interactive bonding” in the literature [32]. Higher catalytic performances can be achieved by combining metal oxides with materials such as carbon nanotubes (CNT), graphene oxide [33], and metal nanoparticles [29,30,34–37].

Vanadium-based oxides are widely used in a variety of applications by virtue of their certain favourable properties including high electrochemical activity, good interaction between molecules or ions, environmental harmlessness, and exhibition of strong electron-electron interactions [38]. Due to their variable oxidation states, their theoretical specific capacitance is considerably higher compared to other metal oxides. Vanadium displays 4 common oxidation states between +5 and +2, each of which can be distinguished by its color. The vanadium oxide forms are VO, VO_2_, V_2_O_3_, and V_2_O_5_. The ruthenium has oxidation states ranging from +8 to −2. The ruthenium oxide forms are RuO_2_ and RuO_4_. Ruthenium’s different oxidation states and electrochemical reversibility make it suitable for use as a modifier of electrode surfaces and an excellent electron transfer agent [39].

In this study, Au nanoparticles (AuNPs) mixed-valence ruthenium and vanadium oxide (VOx-RuOx) films were fabricated on the carbon nanotube (CNT) modified glassy carbon electrode for the sensitive determination of hydrazine. The surface morphology and chemical composition of this prepared AuNPs/(VOx-RuOx)/CNT/GCE electrode were investigated in detail by several techniques such as SEM, HRTEM, EDX, and XRD. This sensor platform is used for the first time in the literature for the determination of hydrazine. The ternary synergistic effect of Au nanoparticle and mixed-valence metal oxides on the oxidation behavior of hydrazine is optimized in pH 10 phosphate buffer solution (PBS). The studies required for sensitivity and stability were successfully performed and the modified electrode was tested in a real sample application.

## 2. Experiments

2.1. Chemicals

RuCl_3_x.H_2_O (Ruthenium content, 45%–55%) NaVO_3_, HAuCl_4_, N,N-dimethylformamide (DMF), HNO_3_, HCl, H_3_PO_4_, and multiwall carbon nanotubes (MWCNTs) (purity > 95%, length 9 µm, diameter 7–15 nm) were purchased from Sigma Aldrich company. It is used as CNT instead of MWCNT in all electrode names. Hydrazine sulfate salt (N_2_H_4_.H_2_SO_4_) was purchased from Merck company. A 5.0 mM Au^3+^ solution was prepared by diluting the concentrated stock from 0.1 M Au^3+^ solution with 0.1 M HCl. Phosphate buffer solution (PBS) was prepared with 0.1 M H_3_PO_4, _and pH was adjusted with the addition of 3M NaOH solution dropwise. Daily prepared hydrazine solutions were used prior to measurements. Ultra-pure water system (Millipore System Inc.) Milli-Q 18.2 MΩ cm was used to prepare all solutions. High-purity nitrogen gas (N_2_) was used 5.0 and had a purity of 99.999%.

### 2.2. Apparatus

All electrochemical experiments Autolab PGSTAT128N and PGSTAT101 were used as potentiostat/galvanostatic systems. As a triple electrode system, the working electrode (a GCE), the reference electrode (an Ag/AgCl (saturated KCl)), and the counter electrode (a platinum wire) were inserted into the voltammetric cell. To evaporate the solvent in the CNT suspension which was dropped to the electrode surface an IR lamp (general electric 150 watts) was used. Electrochemical impedance spectroscopy (EIS) experiments were carried out by applying electrode potential 0.23V to the solution containing 5 mM [Fe(CN)_6_]^3+/4+^ in the presence of 0.1 M KCl in the frequency range of 0.05 to 30,000 Hz. For surface morphology and chemical composition analysis, scanning electron microscope (SEM) (Zeiss 300VP, and Gemini 500), high-resolution transmission electron microscopy (HRTEM) (JEOL 2100 JEM HRTEM), X-ray diffraction (XRD) PANalytical Empyrean diffractometer (parameters: Cu-K-Alpha1, 1.5406 A°; 45 kV, 40 mA, 2Theta 5–90, step size: 0.0130) devices were used.

### 2.3. Procedure of electrode modification

The carbon nanotubes were activated with acid according to the procedure previously described in the literature [40–42]. A 40 mg of activated CNT was dispersed in 5 mL dimethylformamide (DMF) and a black suspension was obtained by ultrasonication. The surface of the glassy carbon electrode was polished on the cloth with 0.1 µm Al_2_0_3_ slurry. After the CNT suspension was dropped onto the clean and dry GCE, the DMF solvent was evaporated under the IR lamp and the electrode was named GCE/CNT. A 10 mL of 10 mM HCl supporting electrolyte solution containing 4.0 mM RuClx.H_2_0 and 50 mM NaVO_3_ was transferred to the voltammetric cell and purged with N_2_ gases for 5 min. The electrochemical pulse deposition (PD) process was studied with minor modifications to the procedures used to obtain other mixed-valent metal oxides (MnOx-MoO, MnOx-VOx) in our previous publications [31,35,38]. Briefly, the electrode potential is kept at –0.9 V for 5 s and then kept at +0.2 V for 5 s and coated by starting the cycle 100 times (not shown). For the formation procedure of gold nanoparticle, using cyclic voltammetry, 15 cycles of –1.3V/+ 0.7 V were decorated on the surface (VOx-RuOx)/CNT/GCE in 5.0 mM HAuCl_4_ (Figure S1). This electrode was named AuNPs/(VOx-RuOx)/CNT/GCE. Scheme shows the mechanism of hydrazine at proposed electrode.

**Scheme:**
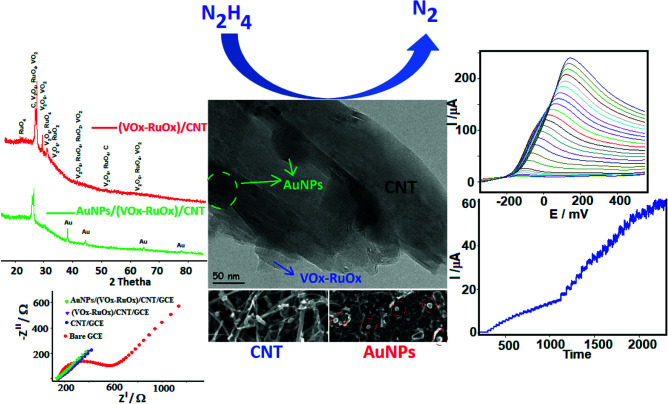
Electrocatalytic mechanism of the oxidation of hydrazine at AuNPs/(VOx-RuOx)/CNT/GCE.

## 3. Results and discussion

The pulsed deposition (PD) technique was used as described in the procedure of formation VOx-RuOx to the surface CNT/GCE (not shown in graphic). In this study, the preparation of VOx-RuOx with PD was performed with minor modification to the procedure of MnOx-VOx, MnOx-MoOx, and MnOx-Vox. used in our previous studies [31,35,38]. Figure S1 shows the cyclic voltammetry (CV) response for the electrochemical deposition of AuNPs on (VOx-RuOx)/CNT/GCE surface. AuNPs were deposited from a solution 5.0 mM Au^3+^ by cycling the potential in the range of –1.3V/+0.7V for 15 cycles. Thus, gold nanoparticles were decorated on the surface of (VOx-RuOx)/CNT/GCE by reducing from Au^3+^ to Au^0^ during the cathodic CV scan [10,43,44]. This electrode was donated as AuNPs/(VOx-RuOx)/CNT/GCE. 

### 3.1. Characterization of modified electrodes 

**Figure 1:**
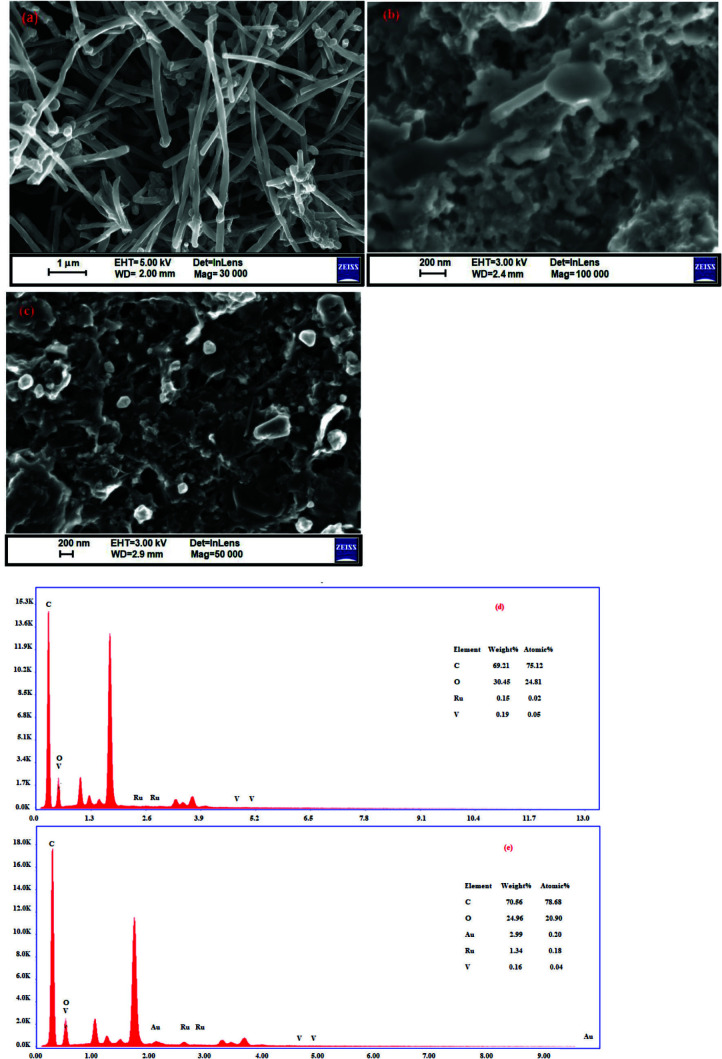
SEM images for (a) bare CNT, (b) (VOx-RuOx)/CNT, (c) AuNP/(VOx-RuOx)/CNT, EDX spectra for (d) (VOx-RuOx)/CNT, (e) AuNPs/VOx-RuOx/CNT.

SEM and HRTEM were used for the characterization of the size and morphology of modified electrodes. SEM images were taken for (VOx-RuOx)/CNT/GCE prepared by using pulsed deposition (PD) on the CNT/GCE from ruthenium and vanadium salts in the same solution. Then, the other electrode (AuNPs(VOx-RuOx)CNT/GCE) was prepared by decorated AuNPs on the (VOx-RuOx)/CNT/GCE with cyclic voltammetry. SEM information of lean carbon nanotubes is given in Figure 1a. Carbon nanotubes are arranged in randomly distributed small tubes on top of each other. As seen in Figure 1b, VOx, and RuOx are uniformly distributed as homogeneous films on carbon nanotube rods. **S** EM images revealed that gold nanoparticles have bright, spherical, and rope ball-like shapes (Figure 1c). The gold nanoparticles were created in scattered nano form and their dimensions were approximately 50 nm. EDX analysis confirms the presence of the expected elements: Ru and V elements in metal oxides, C in the structure of carbon nanotubes, and Au in metal nanoparticles (Figures 1d and e).

**Figure 2:**
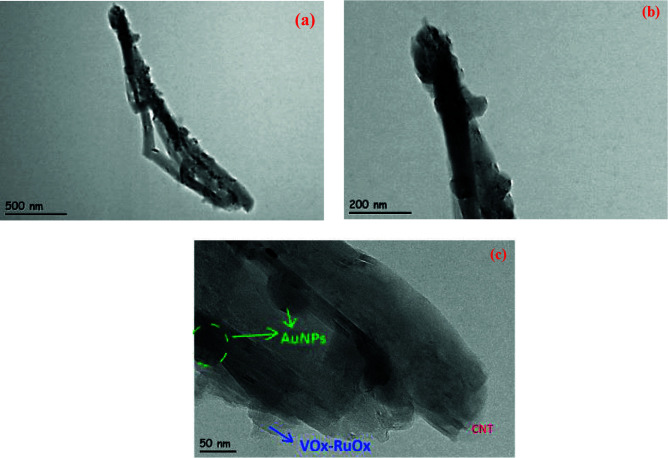
HRTEM analysis for AuNPs/(VOx-RuOx)/CNT(a-c).

HRTEM images of gold nanoparticles onto (VOx-RuOx)/CNT/GCE are presented in Figures 2a-2c. The gold nanoparticles are deposited on the CNT with these metal oxides. The size of the AuNPs is approximately 50 nm and SEM images also support this nanoparticles structure. Moreover, SEM images of mixed metal oxide electrodes revealed that the hierarchical RuOx-VOx oxide layer was distributed evenly by creating a porous cavity between CNT rods. Thanks to this nanopore cavity, a very fast electron transfer hall could be formed from the outside to the inside. Therefore, AuNPs (VOx-RuOx) CNT/GCE has a larger specific surface area, which is an important factor for the electrochemical catalysis. 

**Figure 3:**
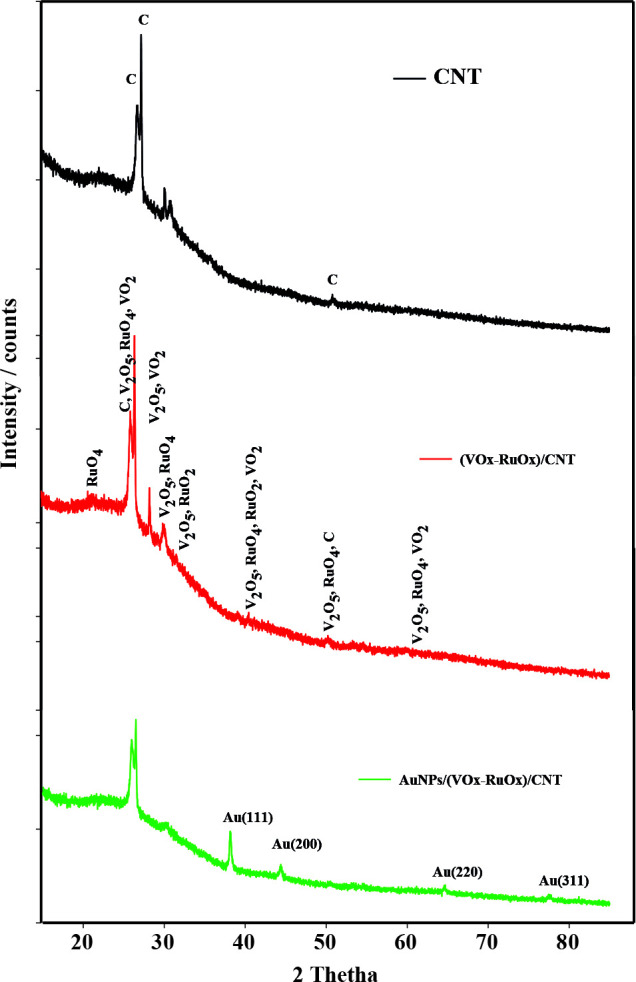
XRD pattern for modified electrodes.

X-ray diffraction (XRD) pattern was applied to identify the crystal structure of modified electrodes (Figure 3). The peak at 2θ = 26.6 ° corresponded with (002) planes in the CNT structure. The crystal structures of VO_2_, V_2_O_5_, RuO_2_, and RuO_4_ were determined from XRD patters by fitting with the ICSD database. The diffraction peaks for VOx were observed at 2θ values of 25.29 °, 28.92 °, 30.92 °, 40.20 °, 49.30 °, and 62.02 °, which correspond to the characteristic diffraction of the planes of monoclinic (ICSD NO 98-000-0199), for V_2_O_5_ observe at 2θ values of 20.31 ^o^, 26.10 ^o^, 31.00 ^o^, 40.20 ^o^, 51.30 ^o^ and 62.17 ^o^, which correspond to the characteristic diffraction of the planes of orthorhombic (ICSD NO 98-001-5984), for RuO_2_ observe at 2θ values of 28.07 ^o^, 40.15 ^o^, 54.34 ^o^, and 65.60 ^o^, which correspond to the characteristic diffraction of the planes of tetragonal (ICSD NO 98-001-5071), for RuO_4_ observe at 2θ values of 23.36 ^o^, 25.62 ^o^, 39.59 ^o^, 49.02 ^o^, and 50.25 ^o^, which correspond to the characteristic diffraction of the planes of cubic (ICSD NO 98-041-5303) [14,45]. We can conclude from XRD results that mixed-valent RuOx and VOx structures occur on the electrode surface. For gold, the peaks observed at 2θ values of 38.20 ^o^, 44.37 ^o^, 64.55 ^o^, and 77.53 ^o^, which correspond to the (111), (200), (220), and (311) of face-centered cubic Au, respectively (ICSD NO 98-004-4362). The characteristic 100% peak is observed at 2θ = 38.18 ^o^ Au(111). This result have been also confirmed by the literature [32,46]. AuNPs on the surface of metal oxides were confirmed as pure metal nanoparticles by XRD.

**Figure 4:**
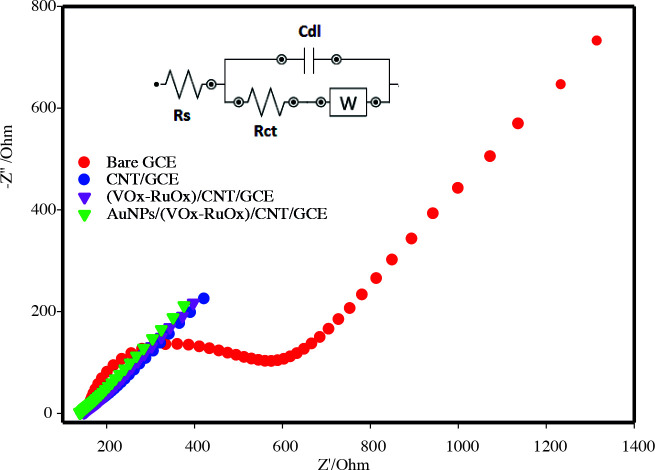
Nyquist curves for modified electrodes.

The electrochemical impedance spectroscopy (EIS) technique is a useful method to evaluate the charge transfer capability at the solution-electrode interface of the synthesized materials. Figure 4 shows Nyquist plots for bare GCE, CNT/GCE, (VOx-RuOx)/CNT/GCE, and AuNPs/VOx-RuOx/CNT/GCE. The diameter of the semicircle obtained by fitting from Nyquist curves gives the electron transfer resistance (Rct) of 14.1Ω for an AuNPs/(VOx-RuOx)/CNT/GCE, 17.2 Ω for a (VOx-RuOx)/CNT/GCE, 30 Ω for a CNT/GCE, and 395 Ω for a GCE. In the results obtained from EIS, the gold nanoparticle modified electrode has the lowest Rct value. This situation can be explained by the fact that gold nanoparticles give the electrode a much more conductive property and increase an active surface area. Also, synergistic interactions between gold and VOx-RuOx on the CNT may play a role in the formation of a porous structure.

### 3.2. Electrocatalytic performance for hydrazine 

**Figure 5:**
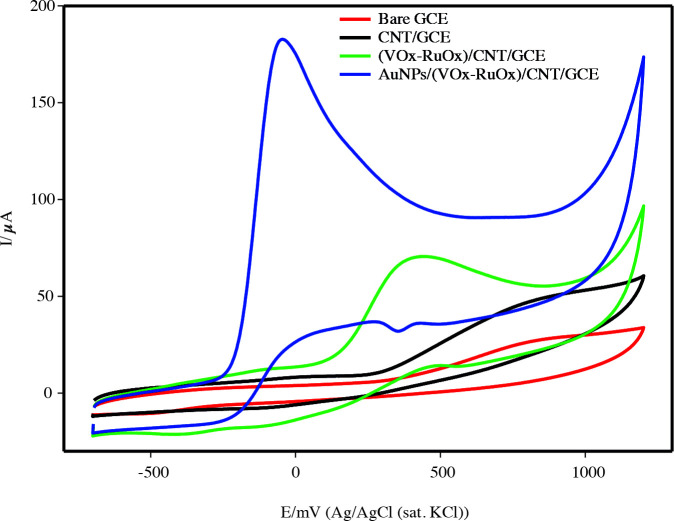
CV responses of 1.0 mM hydrazine on the bare GCE, CNT/GCE, (VOx-RuOx)/ CNT/GCE and AuNPs/(VOx-RuOx)/CNT/GCE in 0.1 M PBS (pH 10.0) with the scan rate of 50 mV s^-1^.

To examine the electrocatalytic activity of the electrodes to the oxidation of hydrazine, cyclic voltammograms were recorded in 0.1 M pH 10.0 PBS saturated N_2_ in different modified electrodes (Figure 5). CVs were performed at a potential scan of –0.7V/ 1.2 V at a scan rate of 50 mV s^–^^1^. Epa and Ipa values of the hydrazine were observed at 800 mV, 20 µA for GCE, 830 mV, 39 µA for CNT/GCE, 433 mV, 55 µA for (VOx-RuOx)/CNT/GCE, and –48 mV, 171 µA for AuNPs/(VOx-RuOx)/CNT/GCE, respectively. A sharp oxidation peak of hydrazine was obtained on the AuNPs/(VOx-RuOx)/CNT/GCE. To reveal the superiority of the modification, the hydrazine current value of the modified electrode was compared with the bare GCE at each step. The oxidation peak flow of hydrazine increased approximately 2-fold in CNT/GCE, 2.7-fold in (VOx-RuOx)/CNT/GCE, and 9-fold in AuNPs/(VOx-RuOx)/CNT/GCE. On the other hand, the hydrazine oxidation peak potential (Epa) at the AuNPs/(VOx-RuOx)/CNT/GCE was shifted to 848 mV in a negative direction (Table S1). The high increase of current in the gold nanoparticle decorated on the electrode may result from an increasingly high specific surface area and a synergistic interaction between Au particles and mixed-valent metal oxide (VOx-RuOx). 

### 3.3. Optimization studies

**Figure 6:**
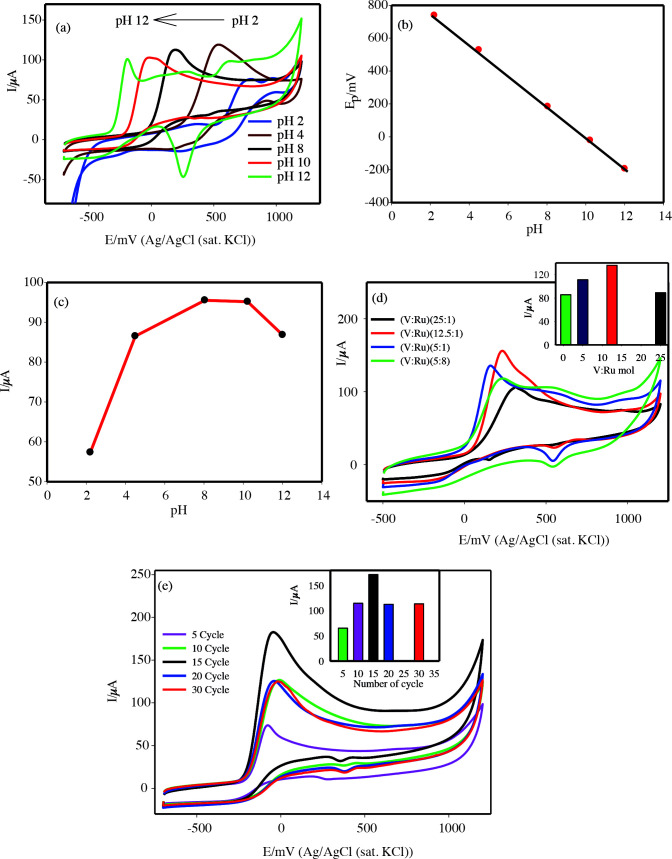
CV responses of 1.0 mM hydrazine on AuNPs/(VOx-RuOx)/CNT/GCE, (a) The effect of pH, (b) Ep vs. pH graph, (c) Ip vs. pH graph, (d) The effect of V: Ru mol ratio, Inset: (Ipa) vs. mol ratio, (e) The effect of the number cycle, Inset: (Ipa) vs. cycles numbers, scan rate of 50 mV s^-1^.

The electrochemical oxidation of hydrazine is known to be affected by pH. Therefore, to investigate the effect of pH of the supporting electrolyte, 1 mM hydrazine was added to the solution at different pHs (2–12 PBS) and CVs were recorded at the AuNPs/(VOx-RuOx)/CNT/GCE (Figure 6a). The oxidation of hydrazine weakly peaked at +700 mV at pH 2. As explained by Hosseini et al., hydrazine is found its protonated form (N_2_H_5_^+^) at a pH value lower than pH = pKa (Pka = 8.1) and hydrazinium ion can be electrostatically repelled by positively charged electrode surface [47–49]. Uncharged form of hydrazine (N_2_H_4_,) is formed after pH 8.0 and an increase in the electrocatalytic peak current was observed after this pH value due to the minimization of the electrostatic repulsion. But when pH is above 12, the peak currents decrease with increasing pH, the changes are attributed to the deprotonation of the hydrazine. In addition, linearity was obtained between pH vs. Ep (Figure 6b), which it can be expressed by the following equations: *E* pa(mV)= –92.8pH + 908.79 (R² = 0.992). Nernstian value used 0.8 ratio of electrons and protons (4e^-^/5H^+^). The slope of Epa vs. pH corresponds to −92.8 mV is very close to the anticipated Nernstian value –80 mV (Eq. 1). Therefore, pH 10 phosphate solution was used as optimum supporting electrolyte for electrocatalytic oxidation of hydrazine at the AuNPs/(VOx-RuOx)/CNT/GCE (Figure 6c). Thus, the overall reaction for the oxidation of N_2_H_4_ can be written as follows due to the participation of protons in the electrode reaction: 

(1)N2H54+4H2O→N2+5H3O++4e-

Hosseini et al. and Dilgin et al. also reported similar results for electrocatalytic oxidation of hydrazine at modified electrode [47,49].

The effect of mol ration V: Ru on the oxidation of hydrazine at the composition AuNPs/(VOx-RuOx)/CNT/GC electrode was investigated. The electrode immersed in the salt solution containing different mole ratios of V: Ru was prepared by the PD technique, and CVs of 1 mM hydrazine were obtained after these electrode surfaces were coated with AuNPS (15 cycles) (Figure 6d and Table S2). The optimum mole ratio of V:Ru was chosen as 12.5:1. To find the catalytic activity of gold nanoparticles, the (VOx-RuOx)/CNT/GC electrode was decorated on the surface in a different cycle number (5–30) at the Au^3+^ solution (Figure 6e). The optimum number of cycles was chosen as 15. It can be deduced from these results that in the prepared electrocatalytic platform, the mol of vanadium in the metal oxide mixture should be 12.5 times greater than ruthenium. On the other hand, the 15-cycle of AuNPs created an ideal synergistic effect for the metal-metal oxide mixture. Due to the increased electrocatalytic activity, a hypo-hyper-d interactive bond can occur on the modified electrode surface. Here it represents nanostructured metal particles (hyper) and metal oxide (hypo).

### 3.4. Effect of scan rate

The effect of different scan speeds on the electro-catalytic performances of the CNT/GCE, (VOx-RuOx)/CNT/GCE, AuNPs/(VOx-RuOx)/CNT/GCE in hydrazine oxidation was examined, and the results are shown in Figure S2. When the square root of the scanning speed increased in all modified electrodes, the oxidation peak current of hydrazine increased in parallel. The oxidation current of hydrazine increased linearly with the square root of the scan speed in AuNPs/(VOx-RuOx)/CNT/GCE and other electrodes. Therefore, it can be suggested that the current on the electrode surface is diffusion controlled.

### 3.5. Sensitivity and selectivity

**Figure 7:**
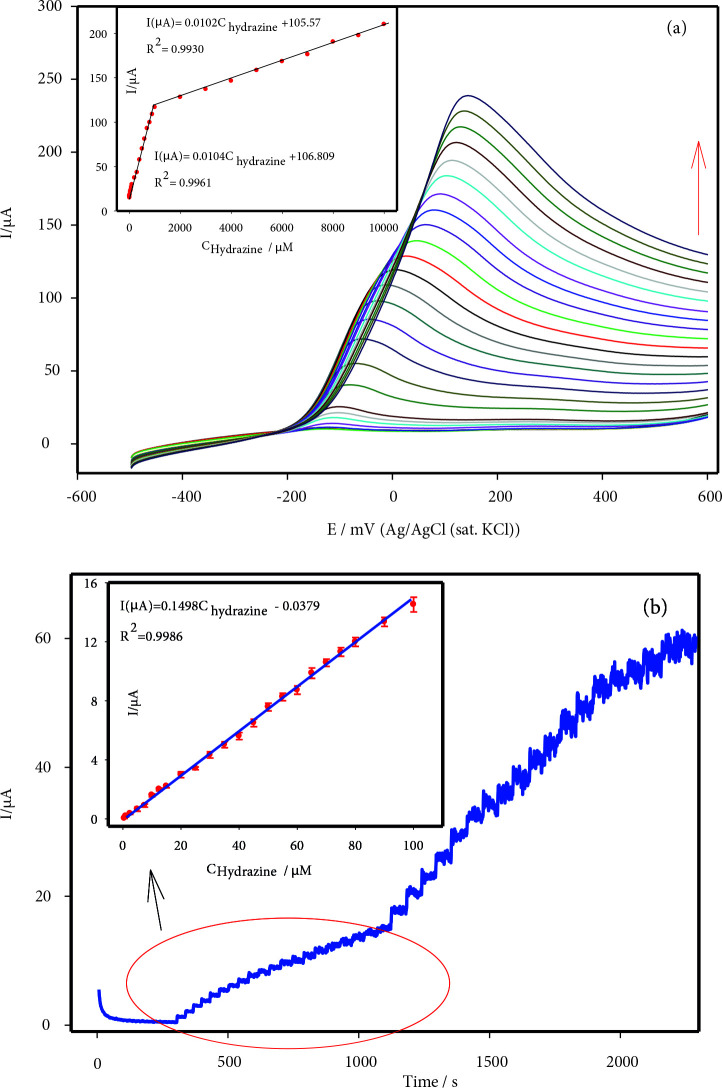
(a) LSVs of various concentrations of hydrazine in pH 10.0 PBS at AuNPs/(VOx- RuOx)/CNT/GCE, (2.5–10 000) μM hydrazine, Inset: calibration graph, (b) Amperomograms for hydrazine (0.3–1000 μM) at AuNPs/(VOx-RuOx)/CNT/GCE at applied potential 0.2 V, Inset: calibration graph (0.3–100 μM).

Linear sweep voltammetry (LSV) and amperometry were used to determine the hydrazine in pH 10.0 PBS at AuNPs/(VOx-RuOx)/CNT/GCE. LSV was studied with a wide range of hydrazine concentrations (2.5–10,000 µM). Also, two different slopes of a linear correlation between oxidation current and hydrazine addition (2.5–1000 µM and 1000–10,000 µM) can be seen in Figure 7a and the inset. LSV reached a peak at –120 mV at low hydrazine concentrations. The oxidation peak shifted to the positive direction potentials, increasing the concentration of hydrazine. The limit of detection (LOD) for hydrazine from LSV was calculated to be 0.50 μM (S/N = 3). 

To determine the concentration of hydrazine more accurately, an amperometric (I-t) response was recorded at AuNPs/(VOx-RuOx)/CNT/GCE at 0.2 V in 0.1 M PBS (pH 10.0) under continuous stirring (Figure 7b). Optimizations of amperometric studies are given in Figure S3. In the amperometric study, the change in the current-time of the modified electrodes was examined, and the highest and stable electrode was the gold electrode at the end of 900s (Figure S4). AuNPs/(VOx-RuOx)/CNT/GCE can respond very quickly to hydrazine addition and a stable signal is received in 4 s. As can be seen in the Figure 7b inset graph, its linearity was obtained from 0.03 µM to 100 µM at the calibration plot. This low value indicates that the electrode can be used for sensitive detection of the hydrazine. The amperometric detection limit (LOD) was calculated as 0.10 μM (S/N = 3).

**Figure 8:**
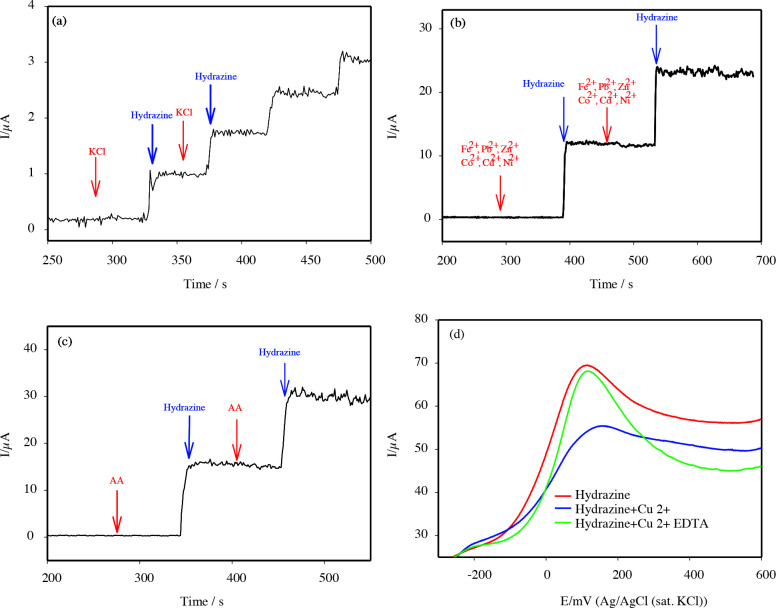
Amperometric graph in the presence of interference added 100 times more than the hydrazine concentration: (a) KCl, (b) Fe^2+^, Pb^2+^, Zn^2+^, Co^2+^, Ni^2+^, Cd^2+^ (Cl^-^, NO^3-^, Cl^-^, NO^3-^, and SO_4_^2-^), (c) AA (ascorbic acid), and (d) the effect of the Cu2+ and EDTA (in the presence or the absence).

Figure 8 shows the amperometric (i-t) response for hydrazine 100-fold KCl, Fe^2+^, Pb^2+^, Zn^2+^, Co^2+^, Ni^2+^, Cd^2+^, Cl^–^, NO_3_^–^, Cl^–^, NO_3_^–^, SO_4_^2–^, and AA (Figures 8a-c). These substances display no significant change of the amperometric currents. A good amperometric response can be observed for hydrazine. However, a significant interference was observed with the addition of Cu^2+^. To eliminate this interference, the linear sweep voltammogram of hydrazine was recorded, the peak current of hydrazine decreased after adding Cu^2+ ^to the same medium (Figure 8d). When excess EDTA was added, the peak current of hydrazine reached its initial value. Thus, Cu^2+^ interference was prevented by creating Cu-EDTA complex in solution.

It can be deduced from these results that the decrease in the peak current of hydrazine in the presence of Cu^2+^ may have caused the interaction between the lone pairs of electrons on the N atom and Cu^2+^. Another reason for this interference may be due to the reduction reaction of Cu (II) to Cu (I) by hydrazine.

To determine the repeatability of the proposed gold electrode, 3 independent electrodes were prepared to detect 1.0 mM hydrazine in pH 10 PBS. A standard deviation of 4.7% was obtained, indicating the reliability of the detection results. Moreover, good stability of the gold electrode and no apparent change of hydrazine oxidation current was observed in the modified electrode after 15 days (Figure S5).

The catalytic activity of the AuNPs/(VOx-RuOx)/CNT/GC electrode to hydrazine detection was compared with previous reports in Table 1. It is clear from Table 1 that the fabricated sensor exhibits the best analytical performance as the LOD and a very wide linear range. 

**Table 1:** Comparison of analytical performances of some hydrazine

Modified electrode	Method	Linear range (μM)	Detection limit (μM)	Ref.
AuNPs/CNTs-ErGO	Amperometry	0.3–319	0.07	[50]
Au@Pt/GO	Amperometry	0.8–429	0.43	[13]
Co3O4/MWCNTs	Amperometry	20–1100	0.80	[51]
rGO-Co3O4@Au	Amperometry	10–620	0.44	[21]
Co3O4/g-C3N4	Amperometry	5–1000	1.00	[20]
NiCo2S4 sphere	Amperometry	1.7–7800	0.60	[8]
Pyrocatechol violet/PGE	FIA Amperometry	0.25–500	0.08	[49]
AuNPs/PGE	FIA Amperometry	0.01–100	0.002	[48]
Au/Choline/GCE	LSV	5–500 500-9300	0.10	[52]
AuNPs/(VOx-RuOx)/CNT/GCE	Amperometry LSV	0.3–1000 2.5–100 100–10000	0.100.50	This work

### 3.6. Application to real sample analysis

To test the practical applicability of the sensor, water samples were collected from the Gediz river and filtered. Gediz river passes from a location close to organized industrial zone in Manisa (Turkey). The hydrazine concentration in these samples was studied using amperometry. The results obtained show a good RSD% of 2.1%–3.2% and reasonable recovery of 97%–108% of hydrazine detection (Table 2). 

**Table 2:** Determination of hydrazine in Gediz river (n = 3).

Sample	Added (µM)	Faund (µM)	RSD (%)	Recovery (%)
1 2 3	0 10 1000	<LOD 9.71 1080	- 2.1 3.2	- 97.1% 108%

## 4. Conclusions

The nanoscale AuNPs/(VOx-RuOx)/CNT/GCE synthesized by electrochemical method, gold-(VOx-RuOx) has favourable properties such as ease of use, high-efficiency, low-cost, greenness, and nonreductiveness. Nanometal particles-metal oxide ternary catalyst was investigated as an electrochemical sensor platform for hydrazine. The electro-catalytic activity of the AuNPs/(VOx-RuOx)/CNT/GCE not only increased the oxidation current of hydrazine but also reduced its oxidation over-potential of hydrazine. Hydrazine is known to be used as a reductant in many industries. Gediz river passes from a location close to organized industrial zone in Manisa (Turkey). The modified electrode developed has been successfully applied to these real samples. Sensitive determinations for hydrazine were obtained by LSV and amperometric methods.
